# A Microfluidic Device to Enhance Viral Transduction Efficiency During Manufacture of Engineered Cellular Therapies

**DOI:** 10.1038/s41598-019-50981-9

**Published:** 2019-10-22

**Authors:** Nathan Moore, John R. Chevillet, Laura J. Healey, Connor McBrine, Daniel Doty, Jose Santos, Bryan Teece, James Truslow, Vienna Mott, Peter Hsi, Vishal Tandon, Jeffrey T. Borenstein, Jenna Balestrini, Kenneth Kotz

**Affiliations:** 1 Biological Microsystems, 555 Technology Square, Draper, Cambridge, MA 02139 USA; 2 Cell and Tissue Engineering, 555 Technology Square, Draper, Cambridge, MA 02139 USA; 3 Synthetic Biology, 555 Technology Square, Draper, Cambridge, MA 02139 USA

**Keywords:** Genetic transduction, Biomedical engineering, Fluid dynamics, Haematopoietic stem cells

## Abstract

The development and approval of engineered cellular therapies are revolutionizing approaches to treatment of diseases. However, these life-saving therapies require extensive use of inefficient bioprocessing equipment and specialized reagents that can drive up the price of treatment. Integration of new genetic material into the target cells, such as viral transduction, is one of the most costly and labor-intensive steps in the production of cellular therapies. Approaches to reducing the costs associated with gene delivery have been developed using microfluidic devices to increase overall efficiency. However, these microfluidic approaches either require large quantities of virus or pre-concentration of cells with high-titer viral particles. Here, we describe the development of a microfluidic transduction device (MTD) that combines microfluidic spatial confinement with advective flow through a membrane to efficiently colocalize target cells and virus particles. We demonstrate that the MTD can improve the efficiency of lentiviral transduction for both T-cell and hematopoietic stem-cell (HSC) targets by greater than two fold relative to static controls. Furthermore, transduction saturation in the MTD is reached with only half the virus required to reach saturation under static conditions. Moreover, we show that MTD transduction does not adversely affect cell viability or expansion potential.

## Introduction

The development and approval of engineered cellular therapies is revolutionizing treatment for an array of diseases that are difficult or impossible to treat with traditional therapeutics. Chimeric antigen receptor T-cell (CAR-T) therapies are proving to be powerful new treatments for difficult to address malignancies^1–3^, while engineered hematopoietic stem cells (HSCs) promise lasting cures for rare blood diseases such as sickle cell anemia, cerebral adrenoleukodystrophy (cALD) and Beta-thalassemia^4–6^. Unfortunately, a major challenge for implementing these treatments is the high cost associated with the complex manufacturing chain, skilled labor, and specialized reagents required for production. These limitations are exacerbated by the fact that the equipment currently used is not explicitly designed for cell-therapy manufacturing^7^. Consequently, a single CAR-T treatment can cost a patient hundreds of thousands of dollars, greatly limiting accessibility and broad use of these treatment modalities^8^. Without significant advancement in bioprocessing technology, scaling up production of these therapies will hinder the wide-scale development of engineered cellular therapies, restricting their use to specific or extreme cases.

The clinically-available cell therapies are autologous products that require the extraction of a targeted cell population from a patient and the genetic manipulation of those target cells to either repair specific genetic defects or enable the target cell to recognize and attack diseased cells within the body^1,2^. Currently, the integration of new genetic material into the target cells is among the most costly and labor-intensive steps in the production of engineered cellular therapies^9,10^. Although both viral and non-viral gene delivery methods, such as sonoporation and electroporation^11–14^, are currently being explored, the most common method for introducing new genetic material into targets cells is through the use of *ex vivo* viral transduction. Conventionally, virus containing packaged genetic material is introduced into the *ex vivo* culture media with target cells under static culture conditions, where gravity and diffusion mediate the colocalization of virus and cell particles. The efficiency of virus particle binding can be modeled using bimolecular first order kinetics of which virus concentration is a significant factor^15^. Centrifugation of target-cell-virus cultures has been demonstrated to increase transduction efficiency, although the exact mechanism for enhanced transduction remains unclear. While evidence has been demonstrated for limited sedimentation of larger HIV-derived virus particles with spin protocols, typical centrifugation speeds are well below those calculated to efficiently sediment virus, particularly smaller viral particles such as adeno-associated virus (AAV)^16,17^. Other explanations for centrifugation-enhanced transduction include stressed induced changes in cytoskeletal structures that favor virus binding, which further suggest that efficacy of centrifugation protocols will vary based on cell stress responses and induction of relevant receptor expression^18^. Alternatively, small-molecule and peptide additives have been developed that bind both virus and target cells, driving interaction between the two particles^19,20^. For example, colocalization of retrovirus and target cells on specific fibronectin fragments increases genetic transduction of mammalian cells by 2–6 fold^21,22^. While these additives have proven to be an effective means of increasing transduction efficiency, most are expensive, proprietary, and must be removed from the final therapeutic product through costly and/or labor intensive washing and validation steps.

By contrast, the use of microfluidics has the potential to effectively drive the colocalization of virus and target cells without the risk of cell damage or the need for extensive product washing^23–26^. Chuck and Palsson demonstrated high rates of viral transduction (total percentage of cells transduced) achieved in relatively short coincubation times when virus-laden media was flowed past target cells trapped against a cell-impermeable membrane^23^. While these methods yielded a high rate of transduction, a significant fraction of virus flows past target cells and through the membrane without interaction, and therefore the efficiency of vector usage (described as the ratio of cells transduced to number of virus particles used) is low, reducing the utility of this method for clinical-scale manufacturing. Alternatively, microfluidic channels have been used to colocalize target cells and concentrated virus in microliter volumes resulting in >4 fold increases in transduction efficiency relative to static controls^24^. Such microchannels work most efficiently at volumes where cells are present at multi-fold higher concentration above typical culture conditions leading to rapid depletion of nutrients and oxygen and limiting the time in which cells can reside in the device. While microchannel technologies have the potential to be effective means of improving transduction efficiencies for cell types with rapid viral binding kinetics, target cells may not respond well to high concentration, prolonged nutrient depletion, or may require longer periods of exposure for effective binding of viral particles. These devices also require pre-concentration of cells with high-titer viral particles, limiting their practical implementation for larger clinical-scale gene therapy.

Here we describe the development and use of a microfluidic transduction device (MTD) that combines microfluidic spatial confinement with advective flow through a membrane to efficiently colocalize target cells and virus particles in order to achieve multi-fold increases in transduction efficiency without damaging target cells. We demonstrate that the MTD can improve the efficiency of lentiviral transduction for T cells by up to 4 fold relative to static controls at sub-saturating multiplicities of infection (MOI). Furthermore, transduction saturation in the MTD is reached with only half the virus required to reach saturation under static conditions. Moreover, we show that MTD transduction does not adversely affect T-cell viability or expansion potential and can be scaled to meet the needs of the cellular bioprocessing field. Finally, we demonstrate the robust performance of the MTD and establish that it can safely improve the transduction efficiency of HSCs.

## Results

### Design and use of the microfluidic transduction device

**Figure 1:**
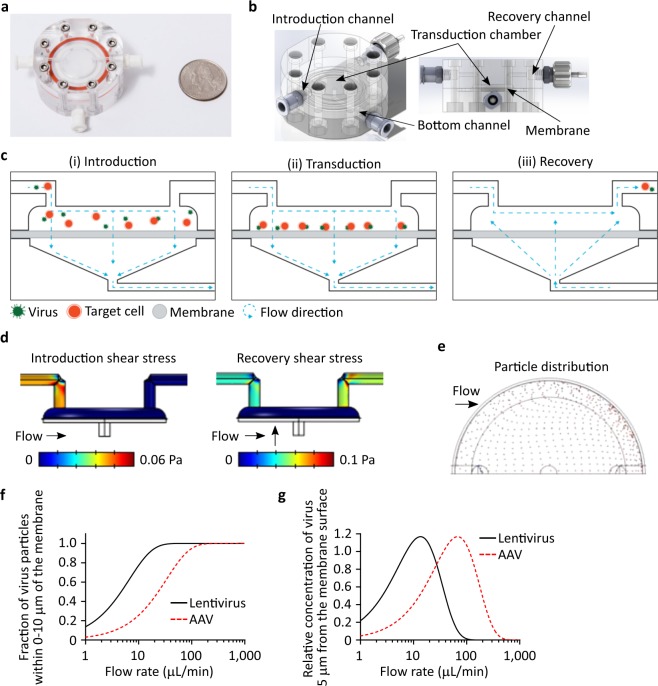
Design of the microfluidic transduction device. (**a**) Photograph of the 2M-MTD (US quarter for size reference). (**b**) Cartoon of the 2M-MTD identifying major design features including the primary transduction chamber and microfluidic flow channels. (**c**) Cartoon for the MTD transduction protocol (i) Target cells and virus are introduced into the device using microfluidic flow down through the semi-permeable membrane, trapping the cells and virus in the transduction chamber. (ii) During transduction, transmembrane fluidic flow pins the cells and virus on the surface of the membrane, increasing the rate of interaction between the two particles. (iii) To recover cells, transmembrane fluidic flow is reversed up through the membrane while additional fluid is pushed across the membrane, driving collection of cells and virus through the recovery channel. (**d**) CFD prediction of wall-shear stresses are low and not expected to negatively impact cell health. (**e**) Top-down view of transduction chamber (only half shown) illustrating CFD predictions of target cell’s point of contract with the porous membrane. The distribution is largely uniform across the membrane surface. (**f**) Analytical model predictions of total relative virus found ≤10 microns of the porous membrane, plotted vs. transmembrane flow rate. (**g**) Analytical model predictions for relative virus concentration in a singular plane 5 microns above the membrane, plotted vs. transmembrane flow rate.

We hypothesize that fluidic flow can be used to improve the colocalization of target cells with viral vector and increase transduction efficiency, thereby reducing vector consumption during the manufacture of engineered cellular therapies. We have devised a scalable microfluidic transduction device (MTD) designed to transduce 2 million (2M) target cells under continuous perfusion in order to colocalize cells and vector. The 2M-MTD is comprised of a semi-permeable membrane sandwiched between two machined acrylic plates and sealed with nested O-rings, creating a membrane-partitioned microfluidic flow chamber (Fig. 1a,b). Introduction and recovery channels within the top plate feed into the transduction chamber positioned above a cell and viral vector impermeable membrane. By design, membranes of various pore size and material can be substituted into the MTD, permitting the use of a broad range of media, target cell, and vector types. The bottom acrylic plate houses a membrane supporting wide-bore nylon mesh and a funneled basin that directs transmembrane fluidic flow into a single bottom outlet channel.

Target cells and virus are introduced into the MTD either in tandem or sequentially by flowing suspension medium into the transduction chamber, down through the membrane, and out the bottom chamber (Fig. 1ci**)**. Computational fluid dynamics (CFD) simulations of fluid flow through the transduction device at operational flow rates predict a uniform distribution of cells and vector across the surface of the membrane with minimal shear stresses (Fig. 1d,e). During the transduction stage (Fig. 1cii), our mass-transport models shows that increasing the transmembrane fluid flow rate increases the fraction of virus at or near the membrane surface (Fig. 1f). Assuming a 100-nm diameter for lentiviral vector (LVV) particles and that all cells are pinned to the surface of the membrane, our model predicts that at a transmembrane flow rate of 20 μl/min, >90% of LVVs will reside in a volume that is within one cell diameter (≤10 μm) from the surface of the membrane. Increasing the flow rate to 75 μl/min will likewise concentrate even smaller viral particles such as AAV. Selecting half a cell diameter (5 μm) as a representative position, our model indicates that a transmembrane flow rate of 20 μl/min maximizes LVV concentration at this position, and increases the concentration by at least 5-fold relative to the concentration predicted for 1 μl/min of transmembrane flow (Fig. 1g). Furthermore, the analytical model predicts a decrease in vector concentration at 5 μm with higher than optimal flow rates as particles concentrate at positions below 5 μm. Therefore, flow rates should be targeted to minimize shear stresses while maintaining maximum transduction efficiency. Perfusion of medium also functions to replenish nutrients and removes waste products, extending the time cells can reside in device relative to other microfluidic transduction systems. Following the transduction period, target cells are recovered by reversing the direction of transmembrane flow while simultaneously applying rapid flow across the transduction chamber, thereby applying low shear to lift cells and direct them toward the recovery outlet (Fig. 1ciii,d). Excess viral vector is subsequently removed by pelleting target cells via centrifugation and aspirating supernatant containing unbound vector.

### The MTD improves transduction efficiency of T cells

**Figure 2:**
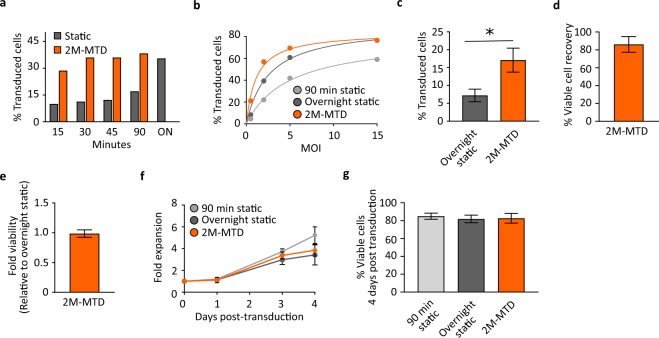
The MTD enhances lentiviral transduction of T cells. CD3^+^ enriched T cells were activated for 48 hours using TransAct reagent and transduced with ZsGreen lentiviral vector in the 2M-MTD. (**a**) Representative experiment for the percent of transduced cells following 2M-MTD or Static transduction of cells at MOI 1.5 for the indicated period of time. ON indicates overnight transduction. MTD reached maximum transduction between 45–90 minutes and was not evaluated overnight for these experiments. Experiment was repeated with similar results. (**b**) Representative data for the percent of transduced cells following static transduction for 90 minutes or overnight and 2M-MTD transduction for 90 minutes at the indicated MOIs. Experiment was repeated with similar results. (**c**) Transduction efficiency for repeat experiments following overnight static transduction or 90 minutes MTD transduction at MOI ~1, n = 8 devices with T cells derived from N = 5 different donors, p < 0.05. (**d**) Percent of T cells recovered from the 2M-MTD with an average of 86%, n = 12 devices with T cells derived from N = 4 different donors. (**e**) T cell viability relative to time matched static controls immediately following removal from the 2M-MTD and washed in media, n = 19 devices with T cells derived from N = 7 different donors. (**f**) Cell counts at the indicated days post-transduction for T cells statically transduced for 90 minute or overnight and T cells transduced in the 2M-MTD for 90 minutes. Cells were transduced with 4 different MOI between 1.5 and 15 and the fold expansion for each transduction condition is shown. (**g**) T cell viability measured by 7AAD and flow cytometry for day 4 post transduction for the same cells in panel f. All error bars indicate standard deviation.

To optimize the use of the 2M-MTD, we first determined the transduction time necessary to obtain maximal lentiviral transduction efficiency of TransAct-reagent-activated CD3-enriched T cells. When T cells were introduced into the 2M-MTD and transduced using lentivirus at a MOI of 1.5 (corresponding to 2–5 fold below saturation levels seen in static conditions), we found that near peak transduction efficiency was achieved with transduction periods between 45 and 90 minutes, well below a single viral half-life of ~6 hours^27^ (Fig. 2a). By contrast, static transduction at 90 minutes was less than half the maximum transduction observed after standard overnight (ON) transduction. Extension of the transduction period for up to 18 hours in the MTD (~3 viral half-lives) led to only marginal improvement in transduction efficiency at this MOI, typically less than 5% of total cells (data not shown).

We next demonstrated that the MTD enhanced transduction efficiencies relative to standard static transduction protocols. When compared to both 90-minute time-matched controls and standard overnight transductions, the 2M-MTD demonstrated improved transduction efficiencies across MOIs ranging from 0.5 to 15 (Fig. 2b). At sub-saturating MOI, the 2M-MTD routinely transduced T cells 4 to 5 fold more efficiently than 90-minute time matched static controls. Moreover, a 90-minute transduction time in the 2M-MTD averaged a 2.4-fold greater transduction efficiency relative to static controls run overnight (Fig. 2b,c). Similar rates of transduction were found to be consistent across data collected using T cells derived from multiple donors (Fig. 2c), and also when we used a second commercially available lentiviral vector (Supplementary Fig. 1a). At higher MOI (~5), the 2M-MTD reached a saturation point in transduction efficiency using approximately half the amount of virus needed with overnight static conditions (MOI between 10 and 15) (Fig. 2b).

The MTD is ultimately envisioned to be a manufacturing tool for therapeutics and it is therefore critical to determine whether the mechanism by which the MTD enhances transduction efficiency biases the transduced population toward particular subpopulations of cells. Although our model and hypothesis predict that the dominant mechanism of action for the MTD is enhanced colocalization of target cell and vector particles, there is a possibility that the device additionally impacts the underlying receptor/coreceptor biology to skew vector binding potential. We therefore examined whether the MTD selectively favored transduction of either the CD4^+^ or CD8^+^ subsets of T cells. Under both static and MTD conditions, transduction rates for CD4^+^ cells were slightly higher than transduction rates for CD8^+^ cells. To determine if the MTD was more selective for either T cell subset, the percentage of transduced CD4^+^ or CD8^+^ cells was normalized to total transduced T cells (CD3^+^). Similar CD4:CD3 and CD8:CD3 ratios between static and MTD transduction conditions were obtained across samples derived from multiple donors and multiple MOIs (Supplementary Fig. 1b,c), suggesting that MTD transduction does not bias transduction towards either CD4^+^ or CD8^+^ T cell subsets. Activation of target cells, causing G_0_ to G_1_ transition and expression of receptors such as LDL-R, is required for efficient static transduction of resting lymphocytes with VSV-G pseudo-typed vector^28–30^. We next asked if the conditions created within the MTD were sufficient to bypass the need for T cell activation and efficiently drive transduction in resting T cells. We observed that transduction of resting T cells remained ineffective using the MTD, with inappreciable rates of transduction in both the MTD and overnight static controls (Supplementary Fig. 1d). These data indicate that activation of T cells is still required for efficient transduction in the MTD.

The benefits of improved transduction efficiency by the MTD are moot if cell recovery is low or if the platform has a deleterious effect on cell viability. To assess the overall health of 2M-MTD transduced T cells, we monitored T cell number and viability immediately following release from the device and for five days post-transduction. On average, 86% of viable cells are recovered from the 2M-MTD with no significant difference in viability relative to static controls (Fig. 2d,e). Furthermore, no statistically-significant differences in cell expansion or viability were observed between 2M-MTD-transduced cells and static transduced controls after multi-day culture, suggesting that transduction in the MTD does not significantly harm or change the expansion potential of T cells (Fig. 2f,g). In combination with the improvement in transduction efficiency, these data demonstrate MTD-mediated lentiviral transduction yields 1.7–3 fold more transduced T cells at sub-saturating MOI relative to cells transduced under static controls, and that transduction saturation can be achieved with half as much virus.

Together, these results demonstrate that the microfluidic device can greatly enhance transduction efficiency without significantly affecting T-cell viability and without the addition of chemical enhancers. Furthermore, the device demonstrates consistent activity across multiple commercially available vectors, suggesting that the MTD is highly adaptable to a range of cell-therapy manufacturing processes flows.

### The MTD can be scaled to meet manufacturing needs

**Figure 3:**
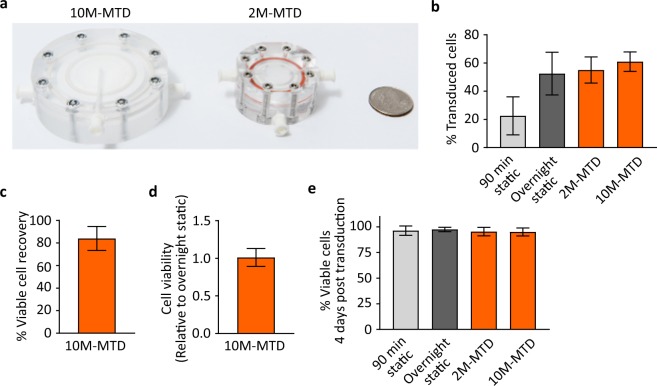
The MTD can be scaled without loss of functionality. 10 million activated T cells were transduced in the 10M-MTD and compared to cells transduced in the 2M-MTD and to static controls. (**a**) Photograph of the 10M-MTD next to the 2M-MTD (US quarter for size reference). (**b**) Transduction efficiency for repeat experiments following 90 minute and overnight static transduction or 90 minutes MTD transduction at MOI of 2.5. (**c**) Average percent of T cells recovered from the 10M-MTD. (**d**) T cell viability relative to static controls immediately following removal from the 10M-MTD and wash in media. (**e**) T cell viability measured by 7AAD and flow cytometry for cells expanded 4 days after transduction. All panels represent data from n = 3 devices with T cells derived from N = 3 different donors. All error bars indicate standard deviation.

Current protocols for the manufacture of engineered autologous cellular therapies routinely require the transduction of 1 × 10^8^ target cells, with some processes even requiring transduction of more than 1 × 10^9^ target cells. To demonstrate that the principles underlying the MTD can scale without losing functionality, we scaled the MTD to accommodate 1 × 10^7^ target T cells (10M-MTD) (Fig. 3a). Overall, the protocol and operations for the loading, transduction, and unloading of cells remained similar to those for the 2M-MTD, with only minor changes in flow rates and cell concentrations to account for the larger membrane surface area. As predicted, the 10M-MTD performed similarly to the 2M-MTD, albeit with a modest increase in T cell transduction efficiency relative to overnight static controls, even at near-saturating MOI (Fig. 3b). Consistent with the 2M-MTD, we recovered an average of 84% of viable T cells from the 10M-MTD and did not observe significant effects on T-cell viability post transduction or following expansion (Fig. 3c–e). These results show that the functionality of the MTD is not diminished when scaled up by one order of magnitude.

### The MTD improves transduction efficiency of HSCs

**Figure 4:**
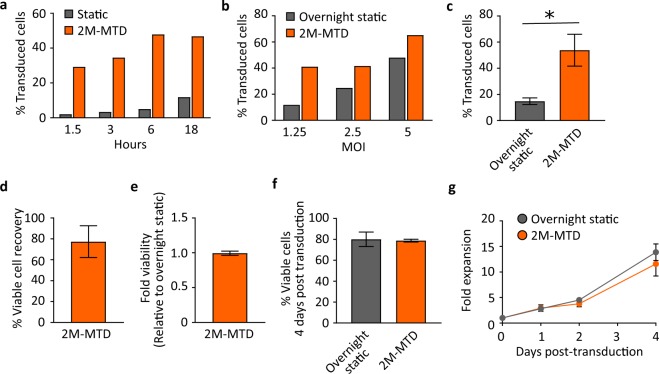
The MTD enhances lentiviral transduction of HSCs. Mobilized CD34^+^ cells were cultured under simulating conditions for 48 hours and transduced with ZsGreen lentiviral vector in the 2M-MTD. (**a**) Representative experiment for the percent of transduced HSCs following static transduction or 2M-MTD transduction at MOI 1 for the indicated period of time. Experiment was repeated with similar results. (**b**) Representative data for the percent of transduced HSCs following overnight static transduction or 2M-MTD transduction for 6 hours at the indicated MOIs. Experiment was repeated with similar results. (**c**) Transduction efficiency following overnight static transduction or at least 6 hours MTD transduction at MOI ~1, n = 4 devices with HSCs derived from N = 2 different donors, p < 0.05. (**d**) Percent of HSCs recovered from the 2M-MTD with an average of 77%, n = 6 devices with HSCs derived from N = 2 different donors. (**e**) HSCs viability relative to matched static controls immediately following removal from the 2M-MTD and washed in media, n = 6 devices with HSCs derived from N = 2 different donors. (**f**) HSC viability measured by 7AAD and flow cytometry day 4 post transduction. (**g**) Cell counts at the indicated days post-transduction for HSCs statically transduced overnight and HSCs transduced in the 2M-MTD for 6 hours. Cells were transduced with at least 3 different MOI between 1.25 and 10 and the fold expansion for each transduction condition is graphed. All error bars indicate standard deviation.

In addition to T cells, an increasing range of cell types including natural killer (NK) and hematopoietic stem cells (HSCs) are actively being evaluated as vehicles for use in engineered cellular therapies^4–6,31^. To further demonstrate the versatility of the MTD, we evaluated in-device transduction of HSCs. In contrast to T-cell transductions, transduction efficiency of HSCs increased in device as the transduction time was increased from 1.5 to 6 hours, but increasing the transduction time to 18 hours did not result in an additional increase in transduction efficiency over the 6-hour time point (Fig. 4a). Strikingly, with as little as 90 minutes of transduction time, we observed an almost 2.5-fold greater transduction efficiency compared to overnight static controls and a near 4-fold improvement at 6 hours. When the MOI was varied, 6-hour transduction rates in device were approximately equal to overnight static controls using twice the amount of virus (Fig. 4b). Together, these results suggest that the MTD can greatly increase the speed of transduction of HSCs while at the same time significantly improving the rate of transduction. Importantly, we observed little donor-to-donor variability, with an average of greater than 3.6-fold improvement in transduction efficiency at MOI 1 across multiple donors (Fig. 4c).

Recovery of viable HSCs from the MTD was 77% on average (Fig. 4d), indicating that when combined with the enhanced transduction efficiency, the MTD yields up to a 2.5-fold net increase in the total number of transduced HSCs. In-device transduction did not significantly impact the viability of the HSCs either immediately following transduction or after 4 days of expansion (Fig. 4e,f**)**. Furthermore, we did not observe significant changes in the rate of HSC expansion post-transduction (Fig. 4g). Together these results demonstrate that the MTD significantly improves transduction efficiency of HSCs without detrimental effects on overall cell health.

## Discussion

Here we have developed a microfluidic transduction device that uses active fluid transport to produce multi-fold enhancement in T-cell and HSC transduction relative to static transduction protocols. We further demonstrated that enhanced transduction efficiency was achieved with a significant reduction in viral vector quantity and target cell coincubation time. Moreover, we demonstrated that processing of cells with the MTD does not significantly affect the viability or expansion potential of either T cells or HSCs post transduction. Importantly, the improvements in transduction efficiency are achieved without the addition of chemical transduction enhancers (e.g. RetroNectin or polybrene), which increase manufacturing material costs and necessitate extra processing steps for their removal during biomanufacturing processes.

Unlike other microfluidic transduction devices, the design of the MTD permits media and nutrient exchange while enhancing efficiency of viral vector usage. Early microfluidic designs by Chuck and Palsson^23^ were able to achieve saturating rates of viral transduction using advection, with transduction efficiency (number of cells transduced relative to amount of virus used) that was ~2.75-fold higher than it was for time-matched static conditions at low viral concentrations. However, as they increased the virus concentration, a greater fraction of the virus particles presumably passed through the membrane without contacting cells, leading to a net increase in vector consumption near saturating concentrations. The MTD achieves a similar increase in transduction efficiencies (~2.6–4 fold relative to overnight and time matched static controls, respectively) at low MOI, but additionally maintains transduction efficiencies greater than or equal to static controls at saturating MOIs by constraining both cell and viral particles near the surface of the membrane, increasing viral vector interaction with target cells without loss of viral particles in the flow stream. Tran *et al*. also achieved 2.5 to 4-fold improvements in transduction efficiencies using microfluidic channels^24^. In their work, Tran and colleagues used geometric constraints related to the microfluidic channels to shorten the diffusion path length for the initial binding of cells with vector. Their data clearly indicate the importance of diffusion and vector-cell binding on the efficiency of transduction. However, microfluidic channel technology requires high initial titers of vector and high cell densities in order to achieve the target transduction efficiencies, which may not always be possible in a high-volume production setting. Furthermore, the scaling of this microfluidic-channel based platform will be limited by challenges with nutrient and gas exchange, as some cell types require extended transduction periods, and therefore will require fresh media perfusion to maintain cell health. By contrast, the design of the MTD automatically concentrates both cell and vector, mitigating challenges associated with low starting viral tiers or low cell densities. In addition, target cells in the MTD are continuously perfused with fresh nutrients and dissolved gases in a highly scalable mechanism that enables at least 18 hours of in-device transduction time.

Our underlying hypothesis and CFD modeling suggest that the primary mechanism of action for improved transduction efficiency with the MTD is driven by enhanced colocalization of vector and target cells, increasing the likelihood of receptor-mediated binding of vector particles to the target cell and promoting viral entry. While virus binding, fusion, entry, and ultimate integration of genetic material is a complex, multi-step progress that is heavily regulated by both cell and virus intrinsic factors as well as extrinsic environmental factors^30,32–35^, binding of viral particles to target cells, which requires contact between the two elements to facilitate viral receptor interactions with ligands on the target cells, has been implicated as a critical, rate-limiting step^15^. Indeed, enhanced binding of vector particles to target cells through microfluidics, centrifugation, and chemical enhancers have all been demonstrated to increase transduction efficiency over relatively short periods of coincubation^20,36,37^. In support of our hypothesis that similar mechanisms drive enhanced transduction in the MTD, we observed that transduction rates of resting T cells remained in the single digits even at high MOI for both static and MTD conditions (Supplementary Fig. 1d). Stimulation of T cells and subsequent upregulation of the vector ligand on the T-cell surface^30^ led to increased transduction in static cells and further enhanced transduction in the MTD (Fig. 2). Together, these data reinforce a dependence on efficient vector ligand expression in the MTD.

We made two key observations regarding transduction in the MTD related to its mechanism of action: (i) For both T cells and HSCs, maximum transduction efficiency is observed for transduction times on the scale of minutes to hours (30–45 min for T cells, and 4–6 hours for HSCs), and increasing the transduction time further does not result in additional increases in transduction efficiency (Figs 2a and 4a), and (ii) the kinetics governing the transduction time required to reach maximum efficiency differs between T Cells and HSCs. There are two factors that may contribute to the dependence of transduction efficiency on incubation time. First, additional incubation time provides time for virus particles to bind to additional cells, but at a rate that diminishes over time due to decay of virus particles in solution and the loss of free virus particles that become bound to cells. Second, additional incubation time may allow for cells to internalize the virus particles, protecting them from becoming unbound during downstream washing steps. Both of these mechanisms are consistent with our observation that increasing coincubation time provides diminishing returns for transduction efficiency. Previous reports have demonstrated that fusion of virus particles to the target cell initiates within 5 minutes and that viral entry time varies between minutes and hours^38,39^. Because these time scales are similar to the optimal incubation times we have observed, we cannot distinguish between the kinetics of virus-cell binding and the kinetics of viral entry, and therefore there remains a possibility that the MTD mechanistically affects both rates.

Interestingly, under similar flow conditions and with the same lentiviral vector, transduction efficiency for T cells reached a maximum in as little as 45 minutes of coincubation time in the MTD, whereas HSCs required 4–6 hours of coincubation time in device to reach their maximum transduction efficiency. Sirvastava *et al*. observed that with HIV, the majority of entry in highly permissive cells occurs within 30 minutes, relative to less permissive cells that required an average of 4 hours^39^. Others have observed cell-type specific viral entry kinetics with delayed vector particle entry in murine hematopoietic progenitors^40,41^. Taken in context with these reports and the lower overall transduction efficiency and longer coincubation of HSCs relative to T cells, our data suggest that cell-type specific viral permissiveness and kinetics associated with viral entry are likely maintained within the MTD. While MTD effects on viral entry kinetics and/or intracellular localization cannot be completely ruled out without further study, our data is well-explained by models based on enhanced vector binding as the dominant mechanism by which transduction efficiency is increased.

Transduction of cells in the MTD results in multi-fold increases in transduction efficiency relative to controls at low MOI (Figs 2b and 4b). As the MOI is increased, the MTD reaches a maximum transduction plateau (saturation) at about half the concentration of virus required for static controls. These results suggest that the MTD provides major benefits for transduction at both low and high MOIs. The key benefit varies based on the operational MOI, and whether transduction is performed below or at saturation along the dose-response curve. In situations where sub-saturating concentrations of virus are used (dilute virus preparation, prohibitive cost of production, imposed limitation on MOI, or safety concerns), the MTD is expected to increase the transduction efficiency and ultimately the total number of transduced cells. In the context of cellular therapies, a larger number of transduced cells greatly reduces the expansion period required to achieve a therapeutic dose, and as cellular therapies are improved and developed, the MTD may even eliminate the need for an expansion period altogether. Alternatively, in cases where vector saturation can be achieved, the MTD is expected to reduce the amount of virus needed to reach transduction saturation by ~50%, while potentially also reducing transduction culture time. As lentiviral vector is a major cost driver in manufacturing engineered cellular therapies, a two-fold reduction in required viral vector has the potential to greatly reduce overall manufacturing costs.

The experiments performed throughout this manuscript are centered around the use of lentiviral vectors to demonstrate the underlying fluidic properties of the MTD to enhance viral transduction. In addition to lentiviral vectors, a broad spectrum of viral vectors, including AAV and retrovirus, are actively being explored in the cell-based therapy market^42–45^. Importantly, colocalization of target cells and virus in the MTD should not be limited to lentiviral based vectors. The MTD is designed to be compatible with the use of a wide range of membranes of varying materials and pore sizes that can be optimized around the use of a desired vector system. Use of membranes with smaller pore diameters (<10 nm) is expected to provide transduction enhancement for AAV-mediated gene delivery similar to that observed for LVVs. Variation of membrane charge or coatings for alternative viral pseudotypes are also straightforward to integrate, and such changes do not require extensive process development^46–48^. Furthermore, transduction flow rates and media can be easily adjusted to meet the physical requirements of the desired target cell and vector system. The flexibility of the MTD therefore enables streamlined integration of the platform into established cell-based therapy manufacturing processes.

## Conclusions

Current equipment and technologies for manufacturing of engineered cell therapies are not explicitly designed for the complex processes and specialized reagents required for production, increasing the challenge of production and exacerbating the high cost and limited patient access associated with these life-saving treatments. In this study, we describe the development and validation of a novel microfluidic transduction device that can be straightforwardly scaled to meet the need for current and future cell bioprocessing throughput while maintaining the flexibility to accommodate variable target cells and viral vectors. We demonstrate that the MTD can safely and consistently improve lentiviral transduction efficiency of T cells and HSCs by up to 4 fold without significantly impacting cell viability or expansion. Furthermore, we demonstrate that optimal transduction efficiency can be maintained with target cell and viral vector coincubation times as short as 45–90 minutes for T cells and 6 hours for HSCs. Deployment of the MTD can lead to a significant reduction in viral vector consumption and potentially reduce overall transduction time. As the integration of new genetic material into the target cells is among the most costly steps in the production of engineered cellular therapies, reducing vector and labor-associated costs will facilitate more wide-scale development of these life-saving treatment modalities and ultimately increase patient accessibility.

## Methods

### Device design, manufacturing, and preparation

The design of the MTD with a circular cavity was developed as a single use device for manufacturability and scalability. Two versions of the MTD were designed to hold 2–6 million cells (2M-MTD) and 4–12 million cells (10M-MTD). The total cell capacity is determined by the surface area of the membrane required to safely hold approximately one monolayer of cells (10 µm) and was designed with a significant error margin to minimize cake layer formation of particles at the surface. We define the fill ratio as the total two-dimensional cross-sectional area of cells to the total membrane area. For the devices in this study, we used a two dimensional fill ratio of 0.6, which is 30% lower than the theoretical maximum hexagonal close-packed monolayer of spheres (0.91). The 2M-MTD and 10M-MTD cell devices were machined from FDA-approved class VI acrylic with a top well depth of 1.6 mm and bottom well depth of 0.5 mm. These depths correspond to the heights of the flow chambers above and below the membrane, respectively. The surface area of the membrane for the 2M-MTD cell device was 250 mm^2^ and the 10M-MTD cell device had a surface area of 512 mm^2^. A polyethersulfone (PES) membrane with 0.03 µm pores (Sterlitech) was compressed between the top and bottom wells, supported by a #50 nylon mesh (McMaster-Carr), and sealed using two nested Viton O-rings (McMaster-Carr). The design was leak tested with both compressed air and a syringe pump and was able to withstand internal pressures greater than 8 bar. Nylon or polycabonate inlet and outlet luer ports (Cole-Parmer) were used to connect to syringes and fluid streams. Some components of the prototype MTDs were reused between experiments. The acrylic plates, O-rings, and luer ports were sonicated in Terg-a-zyme detergent (Sigma Z273287) for at least 30 minutes, thoroughly rinsed in water, and air dried before each use. New membrane and support mesh rounds were used for each experiment. Devices for T cell and HSC transduction were sterilized under ethylene oxide (AN74j Andersen Sterilizers, Inc) or gamma irradiated (>25 kGy VPT Rad or Sterigenics), respectively, prior to use.

### Flow and device modeling

Comsol Multiphysics v5.2 was used to solve the steady-state Navier-Stokes equations for fluid transport in the device. The polymer membrane was modeled as a rigid, porous solid with a thickness 0.150 mm, and the hydraulic conductivity was inferred from the manufacturer’s stated specifications. We used Comsol’s particle-tracing module to calculate trajectories of suspended cells, modeling the cells as point particles with negligible Stokes number. Such cells would thus exactly follow fluid streamlines, unless the streamlines passed through the polymer membrane, in which case the cells would arrest on the membrane surface without slipping. We modeled the distribution of virus near the membrane during the loading phase with the steady-state 1-D convection-diffusion equation, applying a unit bulk-concentration condition far from the membrane and a no-flux condition at the membrane surface. We then scaled the concentration field according to the known total amount of virus in the transduction chamber at steady state. Particle diffusivity was calculated from the Stokes-Einstein equation, approximating the diameter of AAV as 20 nm, and lentiviral vector (LVV) as 100 nm.

### Lentiviral vector titration

Functional titers for the Takara rLV.EF1.ZsGreen1–9 (0038VCT) and SignaGen LV-EF1alpha-GFP-Puro (SL100269) lentiviruses were determined in house using the 293FT (Invitrogen) cell line. In short, 1 × 10^5^ live 293FT cells were seeded into 12-well dishes in 1 mL of culture medium (RPMI 1640 w/10% FBS). Virus stock was diluted 1:625, and then serially diluted by twofold, six times in culture medium. Cells were transduced with 50 µl of each lentivirus dilution in triplicate and mixed gently. ZsGreen or GFP expression was analyzed by flow cytometry five days post-transduction. Functional titers were calculated by taking the negative natural log of the ZsGreen-negative fraction, multiplying by the number of cells transduced (1 × 10^5^) and then normalizing to input volume.

$${\rm{Vector}}\,{\rm{Titer}}=-\,\mathrm{ln}({\rm{fraction}}\,{\rm{ZsGreen}}\,{\rm{negative}})\,\times {10}^{5}\times {\rm{normalized}}\,{\rm{input}}\,{\rm{volume}}$$

### T cell and HSC isolation, activation, and culture

Unpurified, de-identified buffy coat samples containing citrate anticoagulant were purchased from Research Blood Components, LLC and diluted in PBS before Ficoll-Paque (GE Healthcare 17-5442-02) mediated separation of mononuclear cells. T cells were enriched from mononuclear cells using the Miltenyi Pan T-cell Isolation kit (130-096-535) following the manufacturer’s protocol. Isolated T cells were either frozen in Recovery Freezing Medium (Thermo Fisher: 12648010) for later use or placed directly into culture. T cells were cultured in Miltenyi TexMACS medium (130-097-196) in the presence of 100 IU/mL IL-2 (CellGenix 1420-050) and passaged every two-to-three days to maintain 1.0 × 10^6^ cells/mL cultures. T cells were activated with Miltenyi TransAct T-cell Reagent (130-109-104) according to manufacturer’s protocol (111.21D). T cells were cultured under activating conditions for 48 hours before transduction.

Mobilized Peripheral Blood CD34^+^ Stem/Progenitor cells (HSCs) were purchased from AllCells, LLC (mPB017F). HSCs were cultured in SCGM (CellGenix 20802-0500) under cytokine stimulating conditions (Cell Genix: TPO (1417-050), Flt-3L (1415-050), and SCF (1418-050) at 100 ng/mL and IL-3 (1402-050) at 50 ng/mL) and passaged every two-to-three days to maintain concentrations of 1 × 10^6^ cell/mL. HSCs were stimulated for 48 hours before transduction.

Both Research Blood Components and AllCells obtain IRB approved informed consent from all donors giving permission to collect their blood or blood product and use or sell it for research purposes.

### Static transduction

Static transductions were carried out in 24-well culture dishes with a starting density of 1 × 10^6^ cells/mL for both T cells and HSCs in cell-specific culture media. To help ensure equal virus distribution, target cells were pre-mixed with virus particles at the indicated MOI immediately before they were distributed to static control plates or introduced into the MTD. Takara rLV.EF1.ZsGreen1-9 virus was used as the vector for all experiments unless otherwise noted. For transduction time points earlier than 18 hours, cells were collected at the selected time and unbound virus was removed by centrifugation. Virus was not actively removed from overnight transduction samples. Viability and cell number post transduction was measured on a Countess automated cell counter (ThermoFisher). Transduction efficiency was measured by flow cytometry five days post-transduction.

### MTD transduction

MTDs were primed by filling the transduction and bottom chambers with cell-type specific culture media (TexMACS for T cells and SCGM for HSCs) and left to block for at least 15 minutes. Target cells were diluted to 1 × 10^6^ cells/mL (2M-MTD) or 2 × 10^6^ cells/mL (10M-MTD) and pre-mixed with virus before introduction into the MTD. Takara rLV.EF1.ZsGreen1-9 virus was used for all experiments unless specifically noted. Cells and virus were sequestered in the transduction chamber by flowing the suspension through the semi-permeable membrane and out the bottom port via syringe pump pushing at 1 ml/minute. During the transduction period, cytokine-containing medium was perfused across the membrane at a constant rate (20 µl/minute for the 2M-MTD and 40 µl/minute for the 10M-MTD). Cell recovery was achieved by manually pushing media from a syringe across the transduction chamber membrane surface and up through the membrane at an approximate rate of 40 ml/minute. Excess virus was removed from post-transduction cultures by centrifugation of target cells at 300xg for 5 min and aspirating supernatant containing unbound vector. Collected cells were suspended in appropriate culture media and plated in appropriately sized standard culture plates at 1 × 10^6^ cells/ml. To calculate relative cell recovery and viability, recovered cells were counted on a Countess automated cell counter and compared to time and/or condition-matched static controls. Transduction efficiency was measured by flow cytometry four days post-transduction.

### Flow cytometry

PE-CD4 antibody (12-0048-42) was purchased from ThermoFisher. PE/Cy7-CD3 (300420) and BV650-CD8a (301042) antibodies were purchased from Biolegend. Cells for flow analysis were suspended in flow buffer (PBS with 0.5% BSA and 2 mM EDTA) containing antibody and incubated for 15 min at room temperature. Following incubation, cells were washed and suspended in flow buffer containing 7AAD (Thermo Fisher 00-6993-50). Flow cytometry analysis was performed on an Attune Nxt Flow Cytometer (Thermo Fisher) and analyzed using FlowJo V10 software.

### Data collection, figure preparation and statistics

Matrixed experiments of multiple varied conditions using single-replicate data points were performed with single donor and vector lots to map out the relevant parameter space. Multiple donor replicates for specific or optimal conditions were then performed for all reported experiments. Representative data is presented when variability in aggregate data due to differences in the evaluated parameter space, donor variation and/or vector lot variation limit meaningful direct comparison or do not faithfully reproduce the trends observed in individual data sets. Aggregate data sets were used for statistical analysis and include appropriately comparable data points across multiple single-replicate experiment data sets. The number of devices and donors used for aggregate data sets were selected to demonstrate device consistency across donors and ensure adequate statistical power. Graphs were prepared using GraphPad Prism version 8.00 for Windows (GraphPad Software, La Jolla California USA) and figures assembled with Inkscape. Statistical analysis was done using paired t tests in GraphPad Prism.

### Ethics approval and informed consent

The use of human derived cells was reviewed and approved by Draper’s Institutional Biosafety Committee, approval #11.17.2016.1111. All methods were carried out in accordance with the guidelines and regulations approved by Draper’s Institutional Biosafety Committee. All human blood products were purchased from Research Blood Components or AllCells. Both Research Blood Components and AllCells obtain IRB approved consent from all donors giving permission to collect their blood or blood product and use or sell it for research purposes.

## Supplementary information

Supplementary Figure 1

## Data Availability

All data generated or analysed during this study are included in the published article (and its Supplementary Information Files).
