# Supplementary material for: A Microfluidic Device to Enhance Viral Transduction Efficiency During Manufacture of Engineered Cellular Therapies
Source: Sci Rep. 2019 Oct 22;9:15101. doi: 10.1038/s41598-019-50981-9 (PMC6806008; doi:10.1038/s41598-019-50981-9)
Supplement: Supplementary file 1 — Supplementary Figure 1 [file 41598_2019_50981_MOESM1_ESM.pdf]

Supplementary Information

A Microfluidic Device to Enhance Viral Transduction Efficiency During Manufacture of Engineered Cellular Therapies

Nathan Moore*[2], John R. Chevillet[2], Laura J. Healey[2], Connor McBrine[3], Daniel Doty[2], Jose Santos[1], Bryan Teece[1], James Truslow[1], Vienna Mott[1], Peter Hsi[2], Vishal Tandon[1], Jeffrey T. Borenstein[3], Jenna Balestrini[1], and Kenneth Kotz[1]

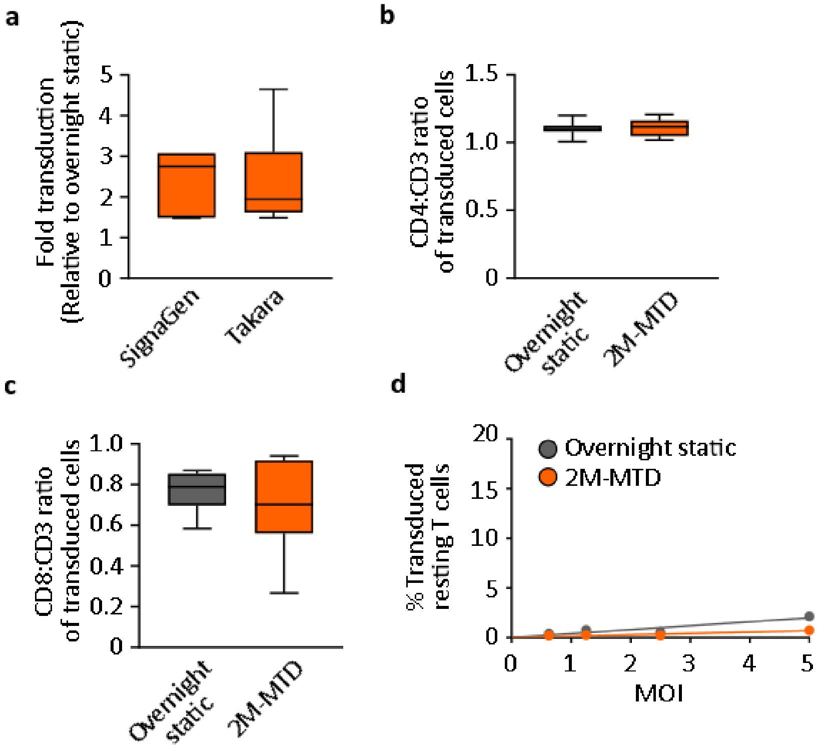

Supplementary Figure 1: Characterization of MTD Enhanced T cell Transduction
(a) Fold change in transduction efficiency for the 2M-MTD using two different commercially available lentiviral vectors. SignaGen average fold increase in transduction of 2.38, n = 7 devices with T cells derived from N = 5 different donors. Takara average fold increase in transduction of 2.38, n = 6 devices with T cells derived from N = 4 different donors. (b) Ratio of $CD4^+$ to $CD3^+$ transduced cells under static and MTD transduction conditions, average ratios of 1.10 and 1.11 respectively, n = 9 devices with T cells derived from N = 6 different donors, p = 0.7. (c) Ratio of $CD8^+$ to $CD3^+$ transduced cells under static and MTD transduction conditions, average ratios of 0.78 and 0.71 respectively, n = 9 devices with T cells derived from N = 6 different donor, p = 0.4. (d) Representative data for the percent transduction of resting T cells following overnight static transduction and 90 minutes transduction in the 2M-MTD at the indicated MOIs.
